# SMAC Mimetic BV6 Induces Cell Death in Monocytes and Maturation of Monocyte-Derived Dendritic Cells

**DOI:** 10.1371/journal.pone.0021556

**Published:** 2011-06-30

**Authors:** Nicole Müller-Sienerth, Lena Dietz, Philipp Holtz, Markus Kapp, Götz Ulrich Grigoleit, Carsten Schmuck, Harald Wajant, Daniela Siegmund

**Affiliations:** 1 Department of Internal Medicine II, University Hospital Würzburg, Würzburg, Germany; 2 Department of Organic Chemistry 2, University Duisburg-Essen, Essen, Germany; University Freiburg, Germany

## Abstract

**Background:**

Compounds mimicking the inhibitory effect of SMAC / DIABLO on X-linked inhibitor of apoptosis (XIAP) have been developed with the aim to achieve sensitization for apoptosis of tumor cells resistant due to deregulated XIAP expression. It turned out that SMAC mimetics also have complex effects on the NFκB system and TNF signaling. In view of the overwhelming importance of the NFκB transcription factors in the immune system, we analyzed here the effects of the SMAC mimetic BV6 on immune cells.

**Principal Findings:**

BV6 induced apoptotic and necrotic cell death in monocytes while T-cells, dendritic cells and macrophages were largely protected against BV6-induced cell death. In immature dendritic cells BV6 treatment resulted in moderate activation of the classical NFκB pathway, but it also diminished the stronger NFκB-inducing effect of TNF and CD40L. Despite its inhibitory effect on TNF- and CD40L signaling, BV6 was able to trigger maturation of immature DCs as indicated by upregulation of CD83, CD86 and IL12.

**Significance:**

The demonstrated effects of SMAC mimetics on immune cells may complicate the development of tumor therapeutic concepts based on these compounds but also arise the possibility to exploit them for the development of immune stimulatory therapies.

## Introduction

SMAC (second mitochondria-derived activator of caspases) / DIABLO (direct inhibitor of apoptosis-binding protein with low isoelectric point) facilitates activation of apoptotic caspases by releasing these proteins from the inhibitory interaction with inhibitor of apoptosis proteins (IAPs), particularly XIAP [1], [2]. SMAC is located in the mitochondria of normal cells, but after permeabilization of the outer mitochondrial membrane in course of apoptosis via the intrinsic pathway, it is released along with proapoptotic proteins such as cytochrome c, which triggers the assembly of the caspase-9 activating apoptosome. SMAC is a dimer and interacts with its four N-terminal amino acid residues (AVPI) with XIAP and the related proteins cIAP1 (cellular inhibitor of apoptosis 1) and cIAP2. In case of XIAP this results in the dissociation of bound caspases and thus easier induction of apoptosis. In case of cIAP1 and cIAP2, SMAC binding triggers autoubiquitination and proteasomal degradation of these molecules [1], [2].

Inappropriate apoptosis resistance is not only of central relevance for the development of tumors per se but also a major factor that drives the evolution of therapy refractory cancer cells. No wonder, there were early on considerable efforts to design molecules that mimic the activity of SMAC to use them in cancer therapy. In fact, in a variety of *in vitro* tumor models, the principle feasibility of this concept has been demonstrated [1], [2]. However, cIAP1 and cIAP2 have also a crucial role in the constitutive degradation of the MAP3 kinase NIK (NFκB inducing kinase) which is an essential signaling intermediate in the alternative NFκB (nuclear factor κB) pathway [3]–[7]. cIAP1 and cIAP2 furthermore contribute to activation of the classical NFκB pathway by a variety of stimuli including TNF [8]–[10]. Treatment of cells with SMAC mimetics therefore regularly results in activation of the alternative NFκB pathway and at least occasionally to some extent also in activation of the classical NFκB pathway. As cIAP1 and cIAP2 in addition antagonize TNFR1-associated caspase-8 activation, SMAC mimetics furthermore sensitize cells for TNF-induced apoptosis [5], [11], [12]. Indeed, SMAC mimetic-induced activation of the NFκB system can result in the up-regulation of TNF which in turn secondarily kills in an autocrine fashion the SMAC mimetic-sensitized cells [4], [5], [13]–[15].

The effects of SMAC mimetics on non-transformed primary cells have been poorly addressed so far. In view of the overwhelming importance of the NFκB system for the regulation of immune cells, we analyzed here the effects of the SMAC mimetic BV6 on human primary immune cells. We found that BV6 induces no or only moderate cell death in most immune cells with exception of monocytes which proved to be quite sensitive. BV6 treatment alone showed furthermore moderate activation of the classical NFκB pathway in dendritic cells (DCs) but diminished the stronger NFκB-inducing effect of TNF and CD40L in these cells. Noteworthy, BV6 treatment was sufficient to drive maturation of monocyte-derived DCs. Taken together, the effects of SMAC mimetics on the immune system may complicate the development of tumor therapeutic concepts based on these compounds but there also arises the possibility to exploit them for the development of immune stimulatory therapies.

## Results

### SMAC mimetic BV6 triggers p100 processing and inhibits TNF-induced signaling

**Figure 1:**
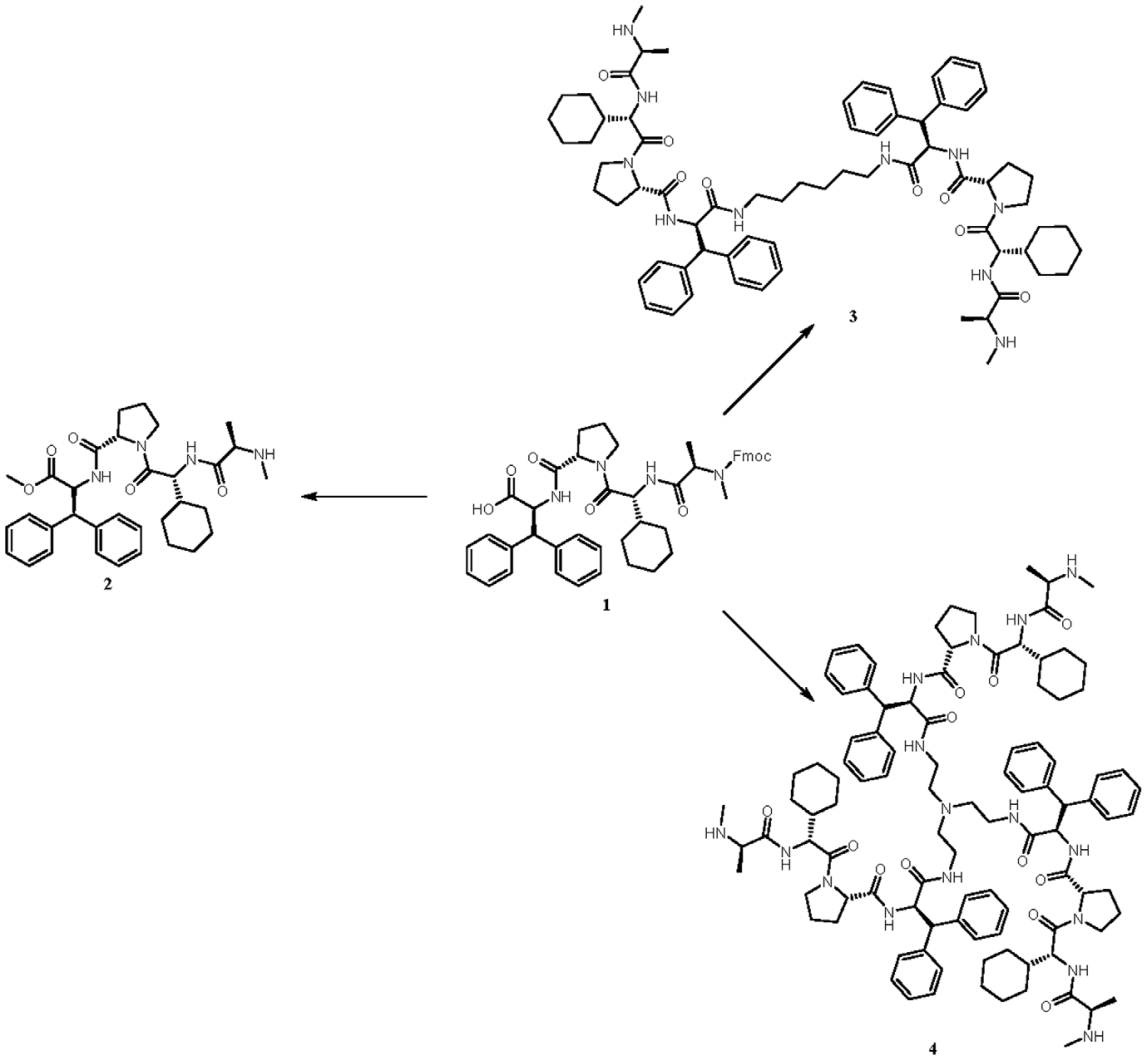
Based on peptide 1 which was synthesized using standard Fmoc solid phase methods on SASRIN™ resin, BV6 (peptide 3) and monovalent (peptide 2) and trivalent (peptide 4) variants derived thereof were synthesized.

**Figure 2:**
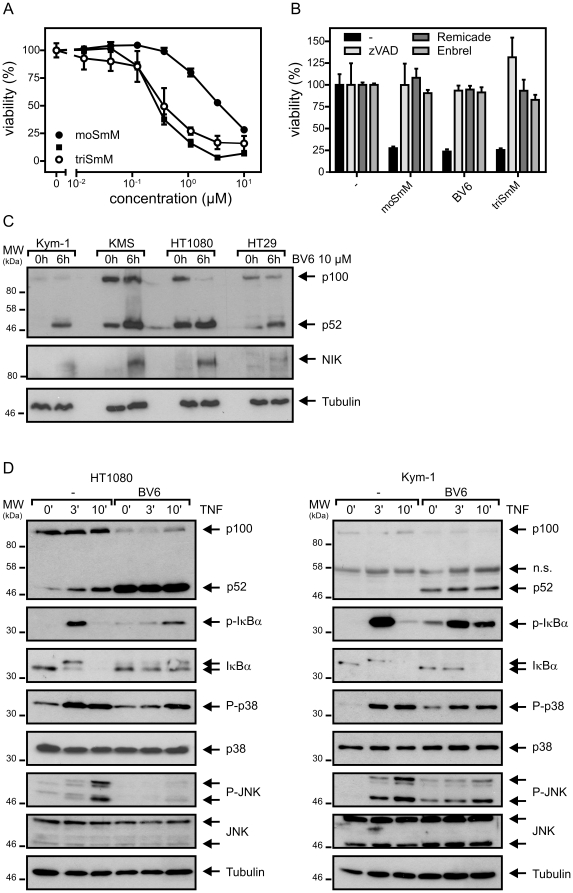
BV6 and its monovalent and trivalent counterparts moSmM and triSmM induce apoptosis in Kym-1 cells and trigger p100 processing in a variety of cell lines. (A) Kym-1 cells were seeded in 96-well plates and stimulated the next day in triplicates with the indicated concentrations of moSmM, BV6 and triSmM. After additional 18 h cell viability was determined by MTT staining. (B), Kym-1 cells were incubated in triplicates overnight (16–18 h) with the indicated mixtures of z-VAD-fmk (20 µM), neutralizing TNF-specific mAb Remicade (40 µg/ml), TNF inhibitor Enbrel (50 µg/ml) and the various SMAC mimetics (10 µM) and viability was then again determined by MTT staining. (C) The indicated cell lines were treated with 10 µM BV6 for 6 hours or remained untreated. Total cell lysates were then analyzed with respect to the presence of the indicated proteins by western blotting. (D) Kym-1 and HT1080 cells were primed with 10 µM BV6 for 6 h or remained untreated and were subsequently stimulated for 0, 3, 10 min with TNF (30 ng/ml). Cells were lysed in Laemmmli sample buffer and analyzed by western blotting for the presence of the indicated molecular species. Detection of phospho-IκBα, phospho-JNK and phospho-p38 is indicative for activation of the classical NFκB pathway and the JNK and p38 MAP kinase cascades. An increase in the p52/p100 ratio further argues for enhanced signaling via the alternative NFκB pathway. Detection of tubulin served as loading control.

We synthesized the recently published bivalent SMAC mimetic BV6 and furthermore generated a monomeric and a trimeric variant derived thereof (Fig. 1). The trivalent variant (triSmM) and BV6 were comparably active while the monovalent compound (moSmM) was ten to twenty-fold less active. This was evident from dose response analyses of SMAC mimetic-induced apoptosis of Kym-1 cells (Fig. 2A,B), which in response to SMAC mimetics undergo apoptosis due to the induction of endogenous TNF [4], [5]. In view of the comparable activity of triSmM and BV6 we used in the following in this study only the bivalent BV6 molecule. The mode of action of BV6 was further confirmed by analyzing p100 processing, a biochemical hallmark of activation of the alternative NFκB pathway, which is an easily verifiable direct consequence of degradation of cIAP1 and cIAP2 [4], [5]. So, in various cell lines including Kym-1 (rhabdomyosarcoma), HT1080 (fibrosarcoma), HT29 (colon carcinoma) and KMS-12-BMS (multiple myeloma), a strongly increased p52/p100 ratio was detectable 6 hours post BV6 treatment (Fig. 2C). There was also accumulation of NIK (Fig. 2C) another well documented consequence of degradation of cIAP1 and cIAP2 [4], [5]. In further accordance with recent data, showing that cIAP1 and cIAP2 also contribute to NIK-independent activation of the classical NFκB pathway in response to TNFR1 by ubiquitination of the TNFR1-associated kinase RIP1 [9], [10], we observed that BV6 attenuates TNF-induced phosphorylation of IκBα (inhibitor of κB-α; Fig. 2D). In accordance with a role of RIP1 ubiquitination in activation of MAP kinase cascades [16], [17], we further observed reduced phosphorylation of JNK and p38 in response to TNF stimulation in BV6-treated cells (Fig. 2D). Although BV6 priming diminished TNFR1-induced phosphorylation of IκBα, JNK and p38, there was nevertheless a slight increase of these responses by BV6 treatment alone (Fig. 2D). Thus, BV6 activates to some extent proinflammatory pathways but the same time interferes with their much stronger stimulation by TNF.

### SMAC mimetic BV6 induces cell death in freshly isolated human monocytes

**Figure 3:**
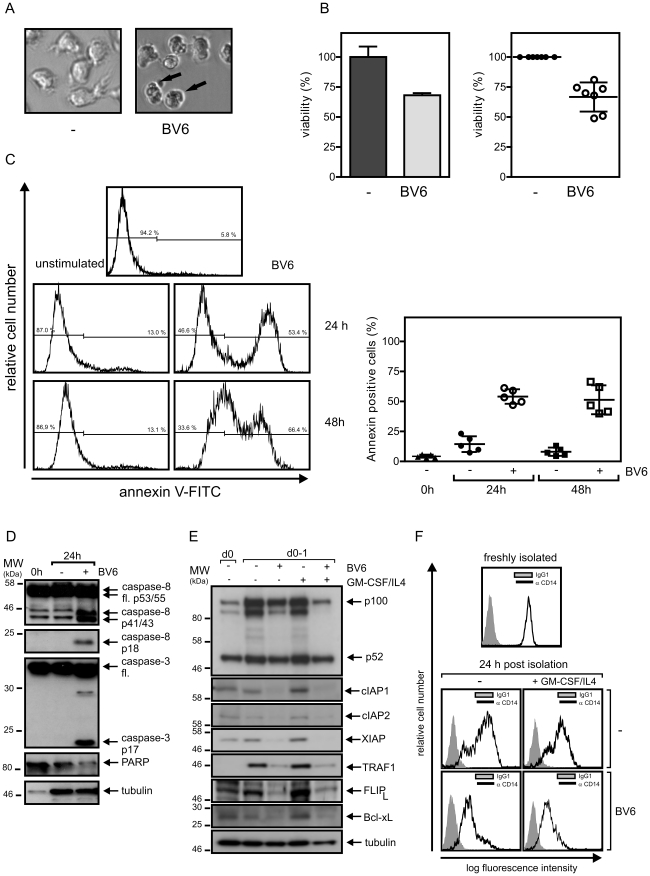
SMAC mimetic BV6 induces cell death in monocytes. (A) Monocytes were isolated from peripheral blood mononuclear cells by MACS separation, cultivated for 1 day in GM-CSF/IL4 supplemented medium in the presence and absence of 10 µM BV6 and finally visually inspected by microscopy. (B) Monocytes were cultivated with 10 µM BV6 for 1 day and viability was assessed using the MTT assay (triplicates, left panel). Viability of BV6 treated cells were normalized against corresponding control samples receiving no BV6 (n = 7, right panel). (C) Effect of 10 µM BV6 on monocyte viability was determined after overnight incubation by annexin-V staining. Left panel shows a representative analysis of one individual sample and the right panel summarizes the data of monocytes independently derived from 5 buffy coat samples. The mean is indicated by a horizontal line. (D) Freshly isolated monocytes (0 h) and monocytes cultivated overnight in GM-CSF/IL4 (24 h) were analyzed by western blotting with respect to the processing of the indicated caspases and PARP-1. (E and F) Freshly isolated monocytes and monocytes cultivated in the presence of the indicated mixtures of GM-CSF/IL4 and 10 µM BV6 were analyzed by western blotting (E) and FACS (F) with respect to the expression of the indicated proteins. The western blot data shown were representative for two – four independent experiments.

To evaluate the effect of BV6 on peripheral blood monocytes, the latter was freshly isolated from buffy coats by ficoll density centrifugation and anti-CD14 magnetic bead separation. Monocytes were then immediately challenged with 10 µM BV6 or remained untreated. MTT analysis and microscopic inspection of monocytes of buffy coat preparations of various independent blood donations revealed that treatment with BV6 strongly reduces the viability of monocytes (Fig. 3A,B). FACS analyses correspondingly showed an increase in annexin-V positive cells from 10–15% to 50–60% (Fig. 3C). Moreover, western blot analyses revealed that there was processing of the initiator caspase-8, the effector caspase-3 and the effector caspase substrate PARP-1 (Fig. 3D). Cultivation of freshly isolated monocytes was accompanied by an upregulation of p100 and the anti-apoptotic proteins TRAF1 and FLIP_L_ (Fig. 3E). Basal expression of cIAP1, cIAP2 and xIAP in monocytes varied between different donors but there was typically a significant upregulation of xIAP upon overnight cultivation (Fig. 3E). Noteworthy, induction of p100, TRAF1, FLIP_L_ and xIAP occurred irrespective of supplementation of the culture medium with GM-CSF/IL4 which we regularly included in the cell culture medium to drive maturation into dendritic cells (Fig. 3E). In accordance with the common mode of action of SMAC mimetics, cIAP1 and cIAP2 were practically not detectable in BV6-treated cells. There was further an almost complete processing of p100. We also noted a strong reduction of xIAP expression in BV6-treated cells (Fig. 3E). This was unexpected as it has been shown that BV6 triggers autoubiquitination and degradation of cIAP1 and cIAP2 but not of xIAP [4]. CD14 expression moderately declined overnight and this was accelerated in BV6-treated cells. Again this effect occurred irrespective whether cells were treated or not with GM-CSF/IL4 (Fig. 3F).

**Figure 4:**
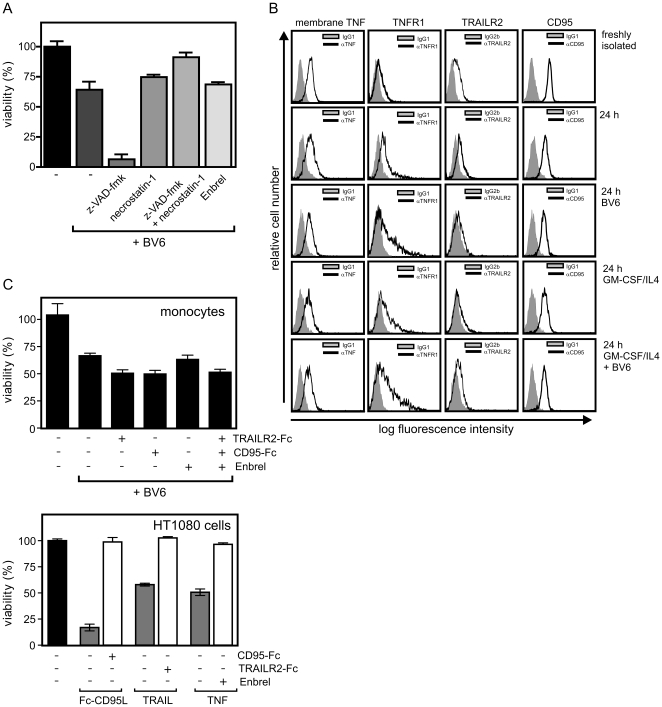
BV6 induces apoptotic and necrotic cell death in monocytes. (A) Freshly isolated monocytes were incubated in GM-CSF/IL4 supplemented medium for 1 h with the indicated mixtures of z-VAD-fmk (20 µM), necrostatin-1 (70 µM) and the TNF inhibitor TNFR2-Fc/Enbrel (50 µg/ml). Cells were then challenged overnight with 10 µM BV6 and cell viability was finally evaluated by help of the MTT assay. (B) Freshly isolated monocytes and monocytes cultivated overnight in the presence of the indicated mixtures of GM-CSF/IL4 and 10 µM BV6 were analyzed by FACS for cell surface expression of membrane TNF and the death receptors TNFR1, CD95 and TRAILR2. (C) Monocytes were challenged with BV6 in the presence of soluble Fc fusion proteins of TRAILR2 (5 µg/ml), CD95 (50 µg/ml) and TNFR2 (Enbrel, 20 µg/ml) or a mixture of them. After 24 h viability was determined using the MTT assay (Left panel). The functionality of the three Fc fusion proteins was controlled in cell death assays with HT1080 and recombinant 50 ng/ml TNF, 4 ng/ml Fc-CD95L and 50 ng/mlTRAIL (right panel). Data shown are representative for three independent experiments.

Next, we evaluated the mechanisms of BV6-induced cell death. The pan-caspase inhibitor z-VAD-fmk showed no protective effect and even resulted regularly in a further reduction of viability of BV6-treated monocytes (Fig. 4A). However, necrostatin-1 an inhibitor of RIP1, which in concert with RIP3 can trigger necrosis [18], attenuated BV6-induced cell death (Fig. 4A). Moreover, when z-VAD-fmk was coapplied with this inhibitor [19], BV6-treated monocytes were almost completely rescued (Fig. 4A). It is well known for TNFR1 and other death receptors that these molecules can induce apoptotic and necrotic cell death dependent on the cell type. As monocytes showed cell surface expression of membrane TNF and TNFR1 (Fig. 4B), we evaluated whether TNFR1 activation by endogenous TNF is involved in SMAC mimetic-induced killing of monocytes. For this purpose, we challenged cells with BV6 in the presence of a high concentration of Enbrel, a TNFR2-Fc fusion protein approved for the treatment of autoimmune diseases such as rheumatoid arthritis. Although, there was a moderate inhibition of cell death induction in some cases, statistical significant protection over all samples analyzed were not achieved (Fig. 4A and data not shown). Thus, sensitization for apoptotic signaling initiated by endogenously produced TNF may contribute to BV6-induced cell death but seems to play no obligate role. As monocytes also express the death receptors TRAILR2 and CD95 (Fig. 4B), we analyzed the effect of BV6 on monocyte viability not only in the presence of Enbrel but also in the presence of Fc fusion proteins of TRAILR2 and CD95. The Fc fusion protein concentrations used were sufficient to rescue HT1080 cells from the apoptotic effect of exogenously applied TRAIL, CD95L and TNF (Fig. 4C). However, there was again no major protective effect even when Enbrel was used in combination with TRAILR2-Fc and CD95-Fc (Fig. 4C).

### SMAC mimetic BV6 induces no or only hardly cell death in monocyte-derived immune cells and T-cells

**Figure 5:**
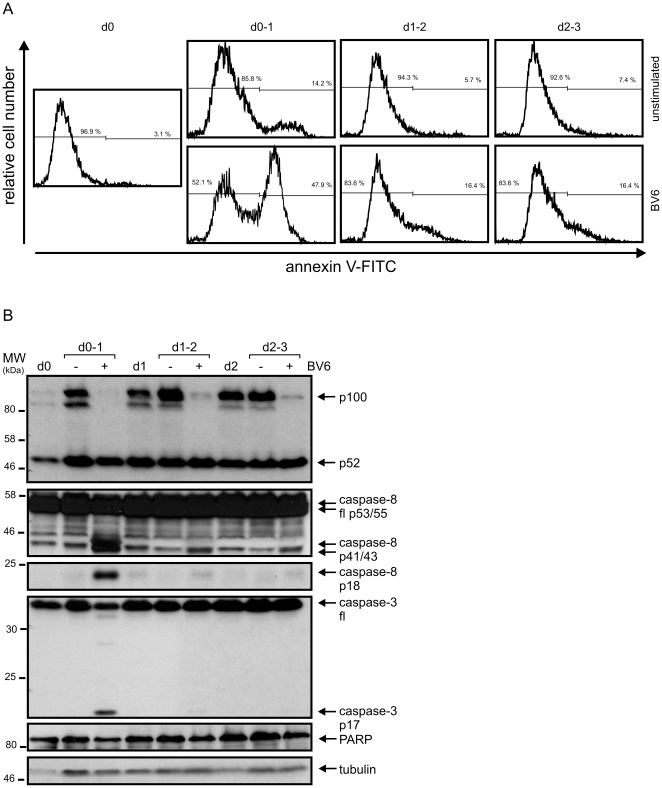
GM-CSF and IL4 rapidly induce resistance against BV6-induced cell death in monocytes. (A and B) Monocytes were cultivated in medium supplemented with GM-CSF/IL4 and were treated immediately or after 1 and 2 days with 10 µM BV6. One day after stimulation BV6 treated cells and a corresponding control sample cultivated without BV6 were analyzed by annexin-V staining (A) and western blotting (B).

**Figure 6:**
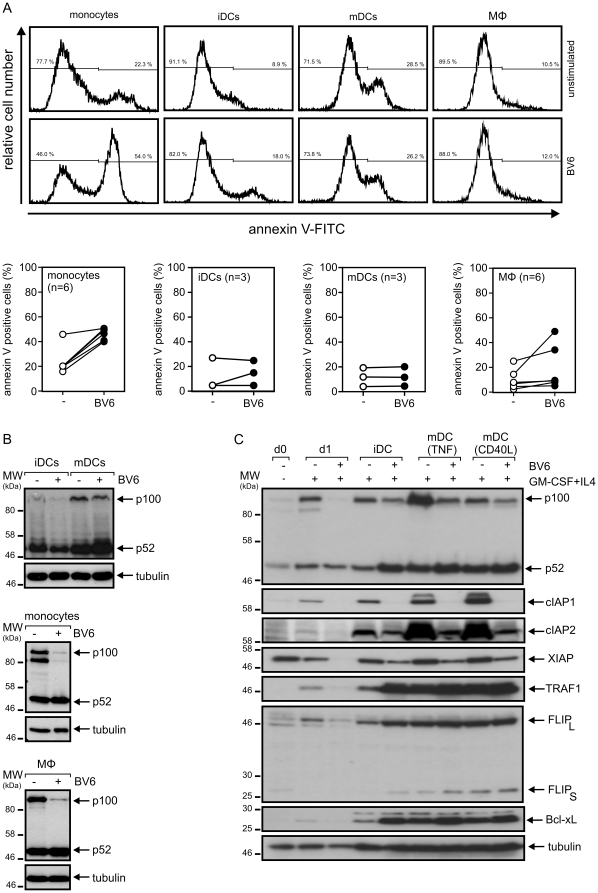
Monocyte-derived dendritic cells and macrophages are barely sensitive for BV6-induced cell death. (A and B) Monocyte-derived macrophages, immature and Fc-CD40L maturated monocyte-derived dendritic cells were challenged for one day with and without 10 µM BV6. Cells were then analyzed by annexin-V staining for cell death induction (A; upper panel: representative analysis of one individual sample; lower panel: summary of the data of 3 independent DC experiments and 6 independent experiments with monocytes and macrophages). p100 processing were determined by western blotting and is shown for one representative experiment (B). (C) Monocytes cultivated overnight in GM-CSF/IL4, iDCs obtained after 7 days of cultivation with GM-CSF/IL4 and mDCs maturated with TNF or Fc-CD40L were analyzed by western blotting with respect to the expression of the indicated proteins (data shown are representative for four independent experiments).

When monocytes were cultured in the presence of GM-CSF and IL4 they rapidly become resistant for BV6-induced cell death. Already one day treatment with these cytokines rendered monocytes almost completely resistant against BV6, although p100 processing was still efficiently triggered (Fig. 5A,B). Moreover, after 7 days of culturing in GM-CSF/IL4 when monocytes were efficiently differentiated into immature dendritic cells (iDCs), they were still poorly killed by BV6 and this also holds true when iDCs were further differentiated with CD40L into mature DCs (mDCs) (Fig. 6A). An alternative route of differentiation of monocytes, which is triggered by M-CSF, results in the generation of macrophages (MΦ). Again, this path of monocyte differentiation was associated with resistance against BV6-induced cell death in 5 of 6 independent samples indicating that MΦ are typically SMAC mimetic insensitive. Western blot analyses of p100 processing indicated that in all cell types treatment with BV6 resulted in the activation of the alternative NFκB pathway (Fig. 6B,C). In view of the inhibitory effect of BV6 on the expression of anti-apoptotic factors in freshly isolated monocytes shown in figure 3E, we also analyzed the expression of IAPs, TRAF1, FLIP_L_ and Bcl-xL in immature DCs and DCs maturated with TNF and CD40L. As expected, expression levels of cIAP1 and cIAP2 were severely reduced in BV6-treated DCs (Fig. 6C). Expression of xIAP was also reduced in BV6-treated DCs but to a much lower extent than in monocytes (Fig. 3E and 6C). Bcl-xL, TRAF1 and cFLIP_L_ are constitutively expressed in iDCs and expression of these molecules was further increased upon maturation with TNF or CD40L (Fig. 6C). More interesting, however, BV6 induced expression of Bcl-xL, TRAF1 and cFLIP_L_ in immature DCs reaching levels comparable to those observed in mature DCs (Fig. 6C). Noteworthy, there was no or only a comparably modest increase in the expression of these molecules in BV6-treated mDCs (Fig. 6C). Thus, apart from the inhibitory effect of BV6 on its direct targets cIAP1 and cIAP2, BV6 elicits opposing effects on the expression of several anti-apoptotic proteins in monocytes and DCs that correlate with the different sensitivity of these cell types for the cytotoxic effect of BV6.

**Figure 7:**
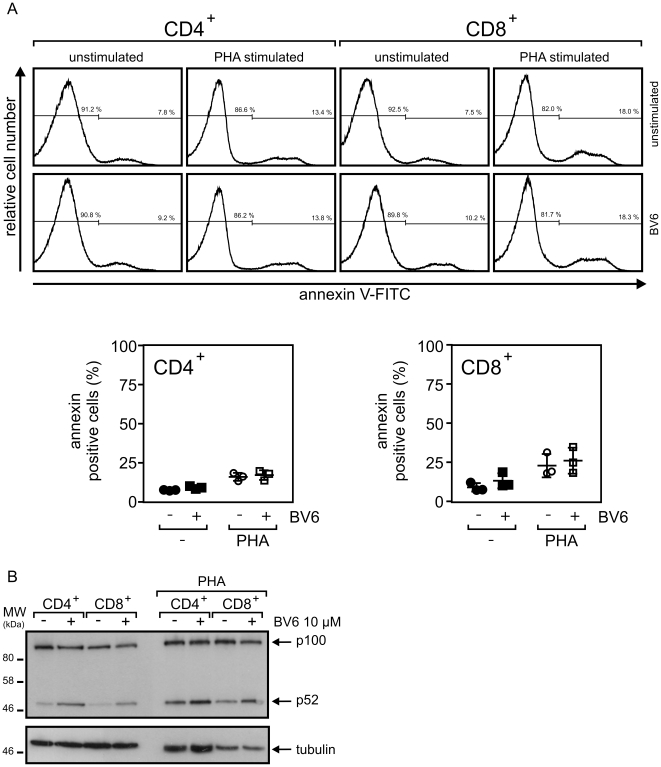
SMAC mimetic BV6 induce no cell death in T-cells. (A and B) CD4^+^ and CD8^+^ T-cells were isolated by magnetic bead separation and were stimulated for 4–7 days with PHA or remained untreated. Cells were challenged with 10 µM or remained untreated as a control and were finally analyzed by annexin-V staining for cell death induction (A; upper panel: representative analysis of one individual sample; lower panel: summary of the data of 3 independent experiments). p100 processing was determined by western blotting and are shown for one representative experiment (B).

Irrespective of their activation status, T-cells isolated from peripheral blood mononuclear cells were also found to be resistant against BV6 (Fig. 7A). In this case, however, BV6 only induced a moderate increase in the ratio of p52 to p100 (Fig. 7B). Taken together, although it appears that BV6 consistently act on the molecular level in immune cells, this may translate only in a subset of these cells in cell death induction.

### SMAC mimetic BV6 triggers alternative NFκB signaling in dendritic cells but inhibits TNF- and CD40L-induced activation of the classical NFκB pathway

**Figure 8:**
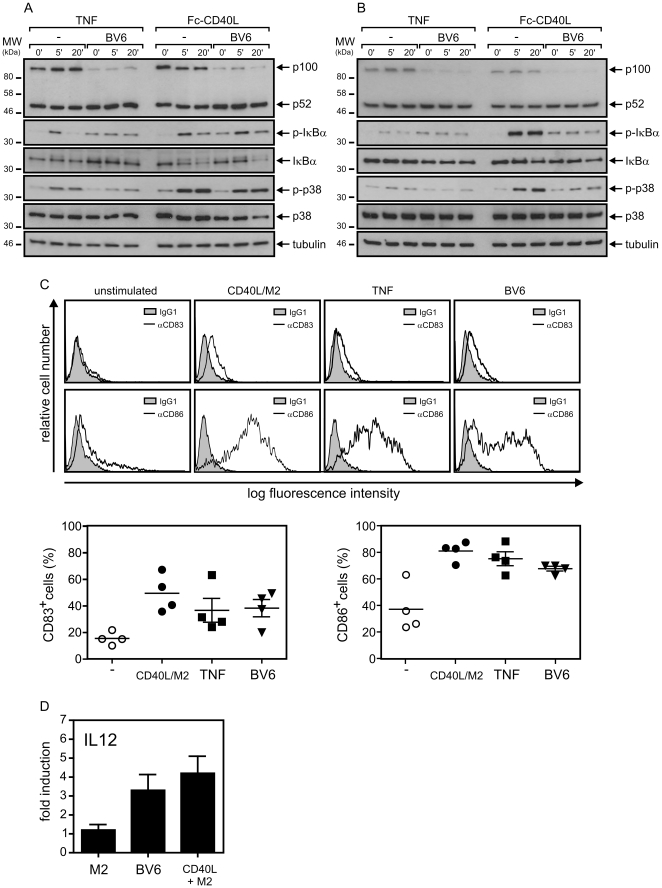
SMAC mimetic BV6 attenuates TNF- and CD40L-induced proinflammatory signaling and triggers DC maturation. (A and B) Immature monocyte-derived dendritic cells (iDCs) were generated by cultivation for 7 days with GM-CSF/IL4. To obtain mature dendritic cells (mDCs), iDCs were further treated with Fc-CD40L (200 ng/ml) for 2 days. mDCs were then cultivated in Fc-CD40L–free medium for additional 24 hours with and without 10 µM BV6 and were stimulated for the indicated times with TNF (200 ng/ml) and Fc-CD40L (200 ng/ml) (A). iDCs were primed overnight with 10 µM BV6 and were then challenged for 5 and 20 min with TNF (200 ng/ml) and Fc-CD40L (200 ng/ml) (B). iDCs and mDCs were finally analyzed by western blotting to determine the presence of the indicated proteins. The results shown with mDCs are representative of three independent experiments, the results with iDCs for two experiments. (C) iDCs were treated with 10 µM BV6, 300 ng/ml TNF and 1 µg/ml of Flag-CD40L oligomerized with 1 µg of the Flag-specific mAb M2 or as a control remained untreated. After three days cells were analyzed by FACS for the cell surface expression of CD83 and CD86. (upper panel: representative analysis of one individual sample; lower panel: summary of the data of iDCs of 4 independent donors. (D) Cell culture supernatants from “C” were analyzed for the presence of IL12. IL12 production was normalized to the corresponding values of untreated cells. The average of experiments with five independent donors is shown.

Next, we evaluated whether BV6 interferes with cIAP1/2-dependent non-apoptotic pathways induced by TNF or the related cytokine CD40L. We thus treated iDCs overnight with BV6 and analyzed the next day TNF- and CD40L-induced phosphorylation of IκBα as an indicator of activity of the classical NFκB pathway. There was hardly detectable IκBα phosphorylation in non-stimulated iDCs, but both cytokines induced after 5 min significant IκBα phosphorylation which was after 20 min already again reduced (Fig. 8A). Priming of the iDCs with BV6 resulted in a significant increase in basal IκBα phosphorylation that almost reached the level of TNF-/CD40L-treated cells. TNF- and CD40L-inducible IκBα phosphorylation, however, was almost completely blocked (Fig. 8A). Similar results were obtained when TNF- and CD40L-induced phosphorylation of p38 was analyzed. Again both cytokines induced a significant response which was attenuated in the BV6-primed cells (Fig. 8A). To further investigate the effects of BV6 on TNF and CD40L signaling in mature DCs, we maturated iDCs with CD40L for three days. Prior analysis of TNF and CD40L signaling in the obtained mDCs, cells were cultivated in CD40L-free medium for one day. Then BV6 were added overnight or not and TNF- and CD40L-induced phosphorylation of IκBα and p38 was analyzed by western blotting (Fig. (8B). The results obtained were essentially similar to those with iDCs. Thus, pretreatment with BV6 again lead to some phosphorylation of p38 and IκBα, but in parallel inhibited the more efficient phosphorylation of these molecules that can be observed in response to TNF and CD40L in untreated mDCs (Fig. 8B).

### BV6 is sufficient to drive maturation of monocyte-derived dendritic cells

A major consequence of p100 to p52 processing is the conversion of p100/RelB heterodimers residing in the cytosol towards p52/RelB heterodimeric complexes that were able to translocate into the nucleus where they stimulate transcription of target genes of the alternative NFκB pathway [20]. Notably, DCs derived from RelB deficient mice are impaired in antigen presentation and T-cell stimulation and the p52 precursor p100 has been furthermore identified as an inhibitor of dendritic cell maturation [21], [22]. We therefore evaluated the effect of BV6 on maturation of monocyte-derived iDCs. We analyzed cell surface expression of the costimulatory molecules CD83 and CD86 that were upregulated in course of DC maturation. 10 µM of BV6 potently induced cell surface expression of both molecules (Fig. 8C). Indeed, upregulation of CD83 and CD86 was almost as efficient as by high concentrations of the well established DC maturation-inducing cytokines TNF and CD40L. Moreover, BV6, like CD40L, also induced IL12 production (Fig. 8D). Due to the strong CD83/CD86 inducing effect of BV6, it was not possible to reliable prove a potential interference with TNF- and CD40L-induced DC maturation.

## Discussion

SMAC mimetics have been developed with the aim to obtain drugs that allow sensitization of tumor cells for apoptosis induction by other drugs or endogenous factors by releasing apoptotic caspases from the inhibitory interaction with IAP proteins, particular XIAP [1], [2]. In fact, several groups reported strong apoptotic effects of SMAC mimetics on tumor cells *in vitro* [5], [11]–[13], [15]. However, the analysis of the mechanisms underlying SMAC mimetic-induced cell death revealed an unexpected mode of action. So, it was found that apoptosis induction was due to the induction of TNF and subsequent stimulation of the death receptor TNFR1 [4], [5], [15]. Notably, most tumor cells are not per se sensitive for TNF and indeed it turned out that SMAC mimetics in addition sensitizes cells for the apoptotic action of TNF [4], [5], [23]. The TNF-related SMAC mimetic effects do not base on XIAP targeting and instead rely on the fact that binding of SMAC mimetics to cIAP1 and cIAP2 induces the proteasomal degradation of these molecules. cIAP1 and cIAP2 are crucial negative regulators of the alternative NFκB pathway [7], but have also an activating role in TNFR1-induced stimulation of the classical NFκB pathway [9], [10]. Furthermore, cIAP1 and cIAP2 inhibit TNFR1-associated activation of caspase-8 [24]. In accordance, SMAC mimetics strongly induce activation of the alternative NFκB pathway and inhibit TNF-induced activation of the classical NFκB pathway ([4], [5], [10], Fig. 2). Nevertheless, SMAC mimetics also induce the classical NFκB pathway to some extent although the underlying mechanisms are unclear but may include crosstalk between the two NFκB pathways or indirect effects of targets induced via the alternative NFκB pathway.

In view of the complex effects of SMAC mimetics on NFκB and TNF signaling and the overwhelming importance of the NFκB system in immune cells, we reasoned that SMAC mimetics also modulate the function of immune cells, an aspect that is certainly of relevance for the development for SMAC mimetic-based therapies. As a first step to a better understanding of the effects of SMAC mimetics on the immune system, we analyzed in this study the effects of the bivalent SMAC mimetic BV6 on various types of human immune cells. First, we evaluated the potential apoptotic effect of BV6. In most types of immune cells namely macrophages, T-cells, immature and mature dendritic cells, BV6 showed typically no significant effect on viability. In freshly isolated monocytes, however, this compound triggered pronounced cell death (Fig. 3). The inability of BV6 to induce cell death in the majority of immune cells was not related to a general “inactivity” of this compound in these cell types because with exception of activated T-cells all immune cells responded with a strong increase in p100 processing to BV6 challenge (Fig. 5B, 6B, and 7B) indicating an inhibitory effect on cIAP1/2. Studies with the pan-caspase inhibitor z-VAD-fmk and the RIP1 inhibitor necrostatin-1 showed that BV6-induced cell death in monocytes base on triggering apoptosis and necrosis (Fig. 4A) pointing to possible effect of TNF induction. Although, there was in some experiments a minor protective effect of TNF-neutralizing reagents, this reached no significance over all samples. Thus, TNF signaling in monocytes seems to play no or only a secondary role in BV6-induced killing of monocytes. We also evaluated the possible implication of other endogenously produced death ligands namely TRAIL and CD95L. Again neutralization of any of these death ligands alone or together with TNF had no major inhibitory effect on BV6-induced cell death (Fig. 4C). However, we observed that BV6 treatment not only down-regulated expression of cIAP1/2 but also the expression of xIAP. The latter was unexpected as BV6 has been described to have no major effect on xIAP expression but could reflect the recent observation that xIAP, cIAP1 and cIAP2 form a complex and stabilize each other [25]. Indeed, it has been shown that xIAP deficiency as well as BV6 treatment sensitize some cells types for cell death induction by the TNFR1-related death receptor CD95 *in vivo* [26]. In view of constitutive expression of membrane TNF on monocytes (Fig. 4B), which allow autocrine or paracrine TNFR1 signaling, one could thus speculate that BV6 sensitizes monocytes for cell death induction by constitutively stimulated TNFR1 and depletion of xIAP. The complex between membrane TNF and TNFR1 might have already be formed prior monocyte isolation and it could be difficult to resolve them afterwards by adding soluble ligand-/receptor-specific blockers explaining the poor protective effect of these compounds in our experiments (see above). So, additional studies in the future must elucidate how BV6-induced cell death operates in monocytes and should finally clarify a possible involvement of TNFR1 or the other death receptors.

BV6 treatment regularly induced cell death only in a fraction (30–50%) of monocytes irrespective of GM-CSF/IL4 treatment (Fig. 3 and data not shown). The only partial response is unlikely to be caused by the failure of some monocytes to respond to BV6 but may rather reflect minor, stochastic differences in cellular activities of survival and cytotoxic molecules. First, upon sensitization of monocytes for necrosis by blocking caspases, BV6 induced cell death in >90% of monocytes treated (Fig. 4A). Second, BV6 triggered accelerated loss of CD14 expression in monocytes surviving BV6 treatment (Fig. 3F). The latter may mirror recent findings demonstrating that cIAP1 knockdown reduces differentiation into macrophages of TPA-treated THP-1 cells and M-CSF-stimulated monocytes [27], [28].

TNF and CD40L are potent triggers of maturation and activation of DCs and utilize cIAPs for the activation of proinflammatory pathways, including the classical NFκB pathway and the p38 MAP kinase cascade. We thus analyzed the impact of BV6 on TNF/CD40L-induced signaling in DCs. Not unexpected in view of the cIAP degrading activity of SMAC mimetics, we found that BV6 priming attenuates the capability of TNF and CD40L to stimulate the activity of p38 and the classical NFκB pathway in immature (Fig. 8A) and mature DCs (Fig. 8B). Noteworthy, BV6 treatment alone led to an increase in the basal activity of the classical NFκB pathway, however, without reaching the strength of the cytokine-induced transient response (Fig. 8A,B). More important, BV6 again induced p100 processing in DCs and in accordance with then known inhibitory role of p100 in DC maturation [22] this was sufficient to drive strong DC maturation *in vitro* (Fig. 8C). Similar to TNF- and CD40L-driven maturation of DCs, BV6-triggered DC maturation was accompanied by an upregulation of NFκB-regulated anti-apoptotic factors including FLIP_L_ and Bcl-xL (Fig. 6C). As BV6 stimulates the classical and the alternative NFκB pathway in DCs and as there can be crosstalk between both pathways, future studies going beyond this initial report must decipher the precise contribution of the two NFκB pathways to BV6-induced DC maturation. It is hardly possible to predict the long term and detailed effects of SMAC mimetics on DC activity *in vivo* only based on *in vitro* data. However, it is tempting to speculate that the DC maturation-inducing, potentially immune-stimulatory effect of BV6 could synergize with its established proapoptotic effect on tumor cells in cancer therapeutic concepts. In fact, while this manuscript was in preparation a costimulatory effect on T-cells and enhanced anti-tumor immunity were demonstrated for the SMAC mimetic LBW-242 [29].

In sum, we noticed four effects of BV6 on immune cells. Firstly, apoptosis induction in monocytes, secondly activation of the alternative NFκB pathway in all investigated immune cells, thirdly enhanced basal but reduced cytokine inducible activation of the classical NFκB pathway in dendritic cells and fourth a potent maturation-inducing activity on DCs.

The diverse functions and the complex interplay of the various cell types of the immune system make it difficult to speculate about the consequences of SMAC mimetics *in vivo* in a conclusive fashion. In view of the aim to use SMAC mimetics in cancer therapy, however, it is evident that effects of SMAC mimetic on the immune system must be considered especially in case of long term treatment. Moreover, there may be even the chance to exploit the immune regulatory effects of SMAC mimetics, e.g. for immunotherapy of cancer.

## Materials and Methods

### Ethics Statement

Macrophages and dendritic cells have been differentiated from monocytes isolated from rests of blood buffy coats of fully anonymized donors obtained from the Institute of Clinical Transfusion Medicine and Hämotherapy of the University Hospital Würzburg and required no special written informed consent. Peripheral blood samples for isolation of PBMCs were obtained after written informed consent (study no. 15/06 approved by the ethic commission of the Medical Faculty of the University of Würzburg).

### SMAC mimetic synthesis

We synthesized the recently published bivalent SMAC mimetic BV6 and monomeric and trimeric variants thereof by methods modified from those used by Vucic et al [4]. In brief, peptide **1** (Fig. 1) was synthesized from commercially available amino acids using standard Fmoc solid phase methods on SASRIN™ resin. Methyl esters were introduced using thionylchloride and methanol. All coupling reactions of peptide **1** to spacers have been done with HATU and diisopropyl ethyl amine. Fmoc protecting groups have been removed with piperidine in DMF. All compounds have been purified using MPLC techniques running methanol/water gradients containing 0.5% TFA. Structure and purity of the various compounds were finally analyzed by NMR, mass spectroscopy and HPLC.

### Isolation of monocytes and differentiation of dendritic cells and macrophages

Monocytes were isolated from blood buffy coats by density centrifugation with LSM 1077 lymphocyte separation medium (PAA Laboratories, Pasching, Austria) and subsequent separation with anti-CD14 conjugated magnetic beads and a MidiMACS Separator (Miltenyi Biotec, Bergisch Gladbach, Germany) following the manufacturer's recommendation. Purity of monocytes (CD14^+^ cells) was controlled by FACS and was regularly >95%. Monocytes were resuspended in RPMI-1460 supplemented with 10% fetal calf serum (FCS), 1% penicillin/streptomycin (PenStrep) (all PAA), 10 ng/ml IL4 (ImmunoTools GmbH, Friesoythe, Germany) and 50 ng/ml GM-CSF (ImmunoTools GmbH) and immediately prepared for the various experiments. For analysis of cell viability using the MTT assay 0.5×10^6^ cells per well of a 96-well plate were seeded in 150 µl medium. For western blot and FACS analysis, 2×10^6^ monocytes per well were seeded in 1 ml in a 24-well plate. Monocytes were differentiated into dendritic cells, by replenishing GM-CSF and IL4 every second day and were used for experiments with immature DCs after 7 days. To obtain mature DCs, iDCs were cultivated for additional three days in the presence of 200 ng/ml Fc-CD40L [30]. Successful differentiation and maturation of dendritic cells were controlled by FACS analysis of CD83, CD86 and CD40 induction. Macrophages were obtained by cultivation of monocytes with 25 ng/ml M-CSF (R&D Systems, Wiesbaden-Nordenstadt, Germany) for 7 days with adding of fresh medium every second day. Differentiation of monocyte-derived macrophages was controlled by FACS analysis of CD14 and CD11b.

### Isolation and treatment of T-cells

PBMCs were isolated by Ficoll-Paque density-gradient centrifugation of cells obtained after plateletpheresis of regular blood donors who gave their written informed consent. CD4^+^ and CD8^+^ T-cells were selected with CD4^+^ and CD8^+^ MicroBeads (Miltenyi Biotech, Germany) and MACS LS columns on the QuadroMACS™ Separator according to the manufacturer's protocol. Purity of CD4^+^ and CD8^+^ T-cells was controlled by FACS and was regularly >95%. CD4^+^ or CD8^+^ cells (3×10^6^) were seeded in a 24-well plate in 1 ml RPMI 1640/10% FCS (Gibco) and were immediately analyzed with respect to BV6-induced cell death or were stimulated with 1 µg/ml phyto-hemagglutinin (Sigma) for 4–7 days to obtain activated T-cells. For analysis of BV6-induced effects, cells were washed three times with PBS to remove any remaining mitogen and were then challenged with BV6 or remained untreated as a control.

### Annexin-V staining and MTT assay

Cells (5×10^5^) were treated as indicated, washed two times in annexin-V staining buffer and were resuspended in 50 µl annexin-V staining buffer supplemented with 1 µl annexin-V solution (ImmunoTools GmbH). After incubation on ice in the dark for 15–20 min, cells were diluted with 100 µl annexin-V staining buffer and analyzed using FACSCalibur (BD Biosciences, Heidelberg, Germany). For determination of cellular viability cells (Kym-1: 2×10^4^ cells per well; monocytes: 50×10^4^ cells per well) were seeded in 96-well plates. Cells were treated as indicated and cell viability was determined using MTT (3-[4,5-dimethylthiazol-2-yl]-2,5-diphenyl tetrazolium bromide; Sigma, Steinheim, Germany) staining.

### Western blot analysis

Cells were washed once in PBS and total cell lysates were prepared by suspending cells (8×10^6^ cells per 100 µl for monocytes, dendritic cells, macrophages and KMS cells; 1×10^6^ cells for Kym-1, HT1080 and HT29 cells) in 4× Laemmli sample buffer (4% SDS, 0.05 M dithiothreitol, 20% glycerol, 0.1 M Tris, pH 8.0) supplemented with phosphatase inhibitor cocktails I and II (Sigma) and complete protease inhibitor cocktail (Roche Diagnostics, Munich, Germany), sonification (ten pulses) and heating for 5 min at 96°C. After clearance of total cell lysates by centrifugation (10 min, 14000 g), samples were separated by SDS-PAGE and transferred to nitrocellulose membranes. Nonspecific binding sites were blocked in Tris-buffered saline containing 0.1% Tween 20 and 5% dry milk and membranes were then incubated with the indicated primary antibodies. After removal of primary antibodies by washing in PBS containing 0.1% Tween 20, antigen-antibody complexes were detected by the help of horseradish peroxidase-conjugated secondary antibodies (Dako, Hamburg, Germany) and an ECL western blotting detection kit (Amersham Biosciences Europe, Freiburg, Germany). Primary antibodies used were specific for caspase-8 (kind gift from K. Schulze-Osthoff; University of Tübingen), caspase-3, NIK, phospho-IκBα, p38, phospho-p38, JNK, phospho-JNK (all Cell Signaling, Frankfurt, Germany), Bcl-xL (S-18) IκBα (Santa Cruz Biotechnologies Inc., Heidelberg, Germany), p100/p52 (Upstate Biotech, Schwalbach, Germany), cIAP2, PARP1 (BD Biosciences Pharmingen, Heidelberg, Germany), cIAP1 (1E1-1-10), FLIP (NF6) (Enzo Life Sciences, Lörrach, Germany) and tubulin (Dunn, Asbach, Germany).
